# Power analysis for genome-wide association studies

**DOI:** 10.1186/1471-2156-8-58

**Published:** 2007-08-28

**Authors:** Robert J Klein

**Affiliations:** 1 Program in Cancer Biology and Genetics, Memorial Sloan-Kettering Cancer Center, 1275 York Avenue, New York, NY, USA

## Abstract

**Background:**

Genome-wide association studies are a promising new tool for deciphering the genetics of complex diseases. To choose the proper sample size and genotyping platform for such studies, power calculations that take into account genetic model, tag SNP selection, and the population of interest are required.

**Results:**

The power of genome-wide association studies can be computed using a set of tag SNPs and a large number of genotyped SNPs in a representative population, such as available through the HapMap project. As expected, power increases with increasing sample size and effect size. Power also depends on the tag SNPs selected. In some cases, more power is obtained by genotyping more individuals at fewer SNPs than fewer individuals at more SNPs.

**Conclusion:**

Genome-wide association studies should be designed thoughtfully, with the choice of genotyping platform and sample size being determined from careful power calculations.

## Background

One goal of modern human genetics is to identify the genetic variants that predispose individuals to develop common, complex diseases. It has been proposed that population-based association studies will be more powerful than traditional family-based linkage methods in identifying such high-frequency, low-penetrance alleles [1]. Such studies require the genotypes a large number of polymorphisms (usually single nucleotide polymorphisms [SNPs]) across the genome, each of which is tested for association with the phenotype of interest. As originally proposed, this would be a direct test of association, in which the functional mutation is presumed to be genotyped. An alternate approach to association studies takes advantage of the correlation between SNPs, called linkage disequilibrium (LD), that can occur due to the genealogical history of the polymorphisms [2]. In this approach, often called indirect association, one SNP is genotyped and used to infer indirectly the genotypes at other SNPs with which it is in high LD [3]. As one genotyped SNP, called a "tag" SNP, can be in LD with numerous other SNPs, much fewer SNPs (10^5 ^– 10^6^) would need to be genotyped to capture the common variation in the genome [3]. Recent advances in genotyping technology make such studies feasible [4,5] and the first results of such studies are being published [6-10].

One key question in designing such studies is the choice of tag SNPs. Numerous methods for choosing the best set of tagging SNPs have been developed and compared [11]. One common measure evaluates the pairwise LD, measured by r^2^, between the tag SNPs and all other SNPs [12]. The value r^2 ^represents the correlation between two SNPs. It is a useful measure because, if N individuals are needed for a specific power with a direct test of association, N/r^2 ^individuals would be needed for an indirect test of association [2]. Sets of tag SNPs are usually compared by their "coverage," or fraction of variants in the genome that are in LD (r^2 ^above some threshold) with at least one tag [12-15].

There are two related problems with this measure of coverage. First, the binary decision of whether r^2 ^is above or below a threshold does not capture the continual decrease in power as r^2 ^decreases. If the cutoff value of r^2 ^is 0.8, a SNP that shows LD of r^2 ^= 0.75 with a tag would be called undetectable since the measure of LD is below the threshold. In truth, association would be detectable, albeit with reduced power. Second, knowledge of the coverage of a set of tag SNPs says nothing about the number of individuals needed for a well-powered study. A better measure to evaluate tag SNPs would be an explicit calculation of the probability that a genome-wide association study will find a statistically significant association given that such an association exists (*i.e.*, power). To solve this problem, one needs to be able to calculate the power of a study given a specified genetic model and sample size. Skol *et al*. have proposed a method for computing power, though they were concerned with issues of study design rather than tag SNP choice [16]. Jorgenson and Witte, who noted the same problems, propose a "cumulative *r*^2 ^adjusted power" that integrates LD and tag SNP information to provide the overall power of a study [17].

Realistically, one does not have an unlimited choice of SNPs but rather chooses among several competing commercial products with fixed sets of tag SNPs. Therefore, instead of choosing a set of tag SNPs, a more common problem now is how to evaluate which of several fixed sets of tag SNPs is better for a particular study. Several papers have looked at power for hypothetical and commercial sets of tag SNPs through empirical simulations on a subset of chromosomal regions [13,18]. This approach suffers from both the speed problem of empirical simulations and the assumption that the sampled regions are representative of the genome as a whole. What is needed is an application of explicit power calculation methods (such as that of Jorgenson and Witte [17]) to the commercially available sets of tag SNPs to allow comparison among products and power calculation for real studies.

Here, I present a method for computing the power of a genome-wide association study when a genetic model and sample size are specified and LD information is available for the population being studied. This method is equivalent to the cumulative *r*^2 ^adjusted power of Jorgenson and Witte [17], which will be referred to as "power" for brevity. I show that to obtain the best power, different commercial genotyping products should be used for different populations. I further find that power is sometimes improved by genotyping more individuals at fewer SNPs rather than fewer individuals at more SNPs. These calculations can guide the optimal design of future genome-wide association studies.

## Results and discussion

The power calculations require genotype data on a large representative sample of common SNPs from the population as well as a list of which of these representative SNPs are the tag SNPs (SNPs to be genotyped). Power is computed in three steps. First the best tag SNP for each of the representative SNPs is found. Then, the power for detecting association for each of the representative SNPs assuming that SNP directly influences the phenotype is computed. For this computation, it is assumed that the study will be performed by testing for genotype frequency differences between cases and controls using a two-degree of freedom *χ*^2 ^test in which multiple tests are corrected for using the Bonferroni correction. This test explicitly assumes a codominant model. I use this test because it is the most general, at the cost of reduced power relative to a model-specific test. While a multimarker tagging approach could be taken [13], this added level of complexity is not usually included in a first-pass analysis of genome-wide association data and is therefore including it in our power-calculation would inflate the power one might expect in real-world application of genome-wide association studies. Finally, the average power over all the SNPs is taken to be the power of the study.

Taking the average power over all the SNPs is justified using probability theory. Assume there are *N *SNPs present in a given population, each one represented as *S*_*i*_. Let *C*_*i *_represent SNP *i *being causative, and *D*_*i *_represent SNP *i *being detected. Assume that one of these SNPs is the causative SNP, but it is unknown which of these is the causative SNP. Then the overall power of the study is given by ∑i=1NPr⁡(Ci,Di)
 MathType@MTEF@5@5@+=feaafiart1ev1aaatCvAUfKttLearuWrP9MDH5MBPbIqV92AaeXatLxBI9gBaebbnrfifHhDYfgasaacH8akY=wiFfYdH8Gipec8Eeeu0xXdbba9frFj0=OqFfea0dXdd9vqai=hGuQ8kuc9pgc9s8qqaq=dirpe0xb9q8qiLsFr0=vr0=vr0dc8meaabaqaciaacaGaaeqabaqabeGadaaakeaadaaeWbqaaiGbccfaqjabckhaYjabcIcaOiabdoeadnaaBaaaleaacqWGPbqAaeqaaOGaeiilaWIaemiraq0aaSbaaSqaaiabdMgaPbqabaGccqGGPaqkaSqaaiabdMgaPjabg2da9iabigdaXaqaaiabd6eaobqdcqGHris5aaaa@3DCE@. The power computed for a specific SNP *S*_*i *_is given as *P*_*i *_= Pr(*D*_*i*_|*C*_*i*_). Thus, if each *P*_*i *_multiplied by Pr(*C*_*i*_), we get

Pr⁡(Ci,Di)=Pr⁡(Di|Ci)Pr⁡(Ci)=PiPr⁡(Ci)Power=∑i=1NPiPr⁡(Ci)
 MathType@MTEF@5@5@+=feaafiart1ev1aaatCvAUfKttLearuWrP9MDH5MBPbIqV92AaeXatLxBI9gBaebbnrfifHhDYfgasaacH8akY=wiFfYdH8Gipec8Eeeu0xXdbba9frFj0=OqFfea0dXdd9vqai=hGuQ8kuc9pgc9s8qqaq=dirpe0xb9q8qiLsFr0=vr0=vr0dc8meaabaqaciaacaGaaeqabaqabeGadaaakeaafaqaaeGabaaabaGagiiuaaLaeiOCaiNaeiikaGIaem4qam0aaSbaaSqaaiabdMgaPbqabaGccqGGSaalcqWGebardaWgaaWcbaGaemyAaKgabeaakiabcMcaPiabg2da9iGbccfaqjabckhaYjabcIcaOiabdseaenaaBaaaleaacqWGPbqAaeqaaOGaeiiFaWNaem4qam0aaSbaaSqaaiabdMgaPbqabaGccqGGPaqkcyGGqbaucqGGYbGCcqGGOaakcqWGdbWqdaWgaaWcbaGaemyAaKgabeaakiabcMcaPiabg2da9iabdcfaqnaaBaaaleaacqWGPbqAaeqaaOGagiiuaaLaeiOCaiNaeiikaGIaem4qam0aaSbaaSqaaiabdMgaPbqabaGccqGGPaqkaeaacqWGqbaucqWGVbWBcqWG3bWDcqWGLbqzcqWGYbGCcqGH9aqpdaaeWbqaaiabdcfaqnaaBaaaleaacqWGPbqAaeqaaOGagiiuaaLaeiOCaiNaeiikaGIaem4qam0aaSbaaSqaaiabdMgaPbqabaGccqGGPaqkaSqaaiabdMgaPjabg2da9iabigdaXaqaaiabd6eaobqdcqGHris5aaaaaaa@6CE9@

The added assumption that each SNP is equally likely to be causative yields

Pr⁡(Ci)=1NPower=∑i=1NPi1N=1N∑i=1NPi
 MathType@MTEF@5@5@+=feaafiart1ev1aaatCvAUfKttLearuWrP9MDH5MBPbIqV92AaeXatLxBI9gBaebbnrfifHhDYfgasaacH8akY=wiFfYdH8Gipec8Eeeu0xXdbba9frFj0=OqFfea0dXdd9vqai=hGuQ8kuc9pgc9s8qqaq=dirpe0xb9q8qiLsFr0=vr0=vr0dc8meaabaqaciaacaGaaeqabaqabeGadaaakeaafaqaaeGabaaabaGagiiuaaLaeiOCaiNaeiikaGIaem4qam0aaSbaaSqaaiabdMgaPbqabaGccqGGPaqkcqGH9aqpdaWcaaqaaiabigdaXaqaaiabd6eaobaaaeaacqWGqbaucqWGVbWBcqWG3bWDcqWGLbqzcqWGYbGCcqGH9aqpdaaeWbqaaiabdcfaqnaaBaaaleaacqWGPbqAaeqaaOWaaSaaaeaacqaIXaqmaeaacqWGobGtaaaaleaacqWGPbqAcqGH9aqpcqaIXaqmaeaacqWGobGta0GaeyyeIuoakiabg2da9maalaaabaGaeGymaedabaGaemOta4eaamaaqahabaGaemiuaa1aaSbaaSqaaiabdMgaPbqabaaabaGaemyAaKMaeyypa0JaeGymaedabaGaemOta4eaniabggHiLdaaaaaa@56C1@

This final equation is the same as taking the average power over all the SNPs.

This method was applied to examine the power of genome-wide association studies in the four populations studied in the International HapMap Project [19]. I examined the performance of the tag SNPs provided by the major high-density genotyping platforms available commercially: 100 K and 500 K SNP sets from Affymetrix and 300 K and 550 K SNP sets from Illumina. (Since then, more products have come on the market; the same approach can be taken with them.) I first asked how many SNPs on each of these arrays would be useful for studying a given population by asking what percentage of tag SNPs provided by each platform are common (minor allele frequency > 5%) in each of the four HapMap populations (Table 1). The largest fraction of common SNPs is found when the Illumina chip is used in the CEU population. As the Illumina chip was designed to optimize coverage of the CEU population, this result is unsurprising.

**Table 1:** The number of SNPs present in each population and present in each commercial genotyping system

Population	CEU	JPT+CHB	YRI
SNPs in HapMap	3868157	3890416	3796934
SNPs w/MAF >= 0.05 (%)	2230515 (58%)	2046163 (53%)	2477182 (65%)
Common SNPs on Affy 100 K chip (%)	91400 (79%)	82995 (72%)	91363 (79%)
Common SNPs on Affy 500 K chip (%)	378415 (77%)	346887 (70%)	409849 (83%)
Common SNPs on Illumina 300 K chip (%)	313265 (99%)	251560 (79%)	252678 (80%)
Common SNPs on Illumina 550 K chip (%)	506543 (91%)	425631 (77%)	441884 (80%)

The percentages given are the fraction of SNPs from the overall SNP set, and from each of the genotyping platforms, that are present with a MAF of at least 0.05 in each population

**Figure 1:**
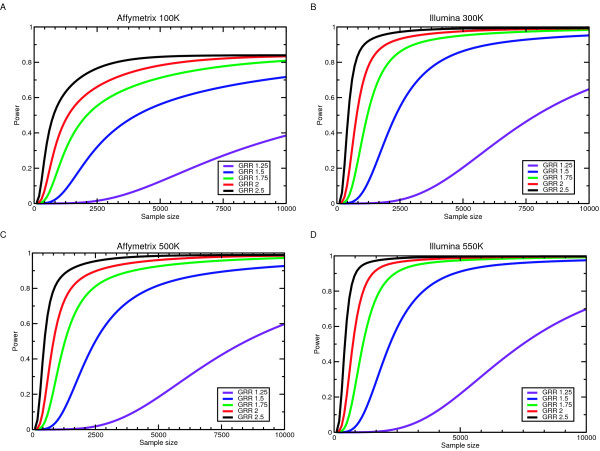
Power for the test of genotypic association as a function of sample size at different genotype relative risks (GRR). All panels are for the CEU HapMap population when the number of cases equals the number of controls and a multiplicative model is used. **(A) **Power for the Affymetrix 100 K system. **(B) **Power for the Illumina 300 K system. **(C) **Power for the Affymetrix 500 K system. **(D) **Power for the Illumina 550 K system.

I next asked how power changes with increasing sample size for the various genotyping platforms (Figure 1), populations, and models. For all sets of tag SNPs, as expected, power increases both as the sample size increases and as the magnitude of effect, measured by the genotype relative risk (GRR), increases. While Figure 1 only shows this data for a multiplicative model in the CEU population, similarly shaped curves were observed in the other populations and for other models [see Additional file 1]. In the Affymetrix 500 K and Illumina 300 K SNP sets, the slope of the power curve starts leveling off (approaching zero) with a few thousand individuals when GRR is more than 1.5. For smaller GRRs, the sample sizes required for adequate (at least 50%) power becomes quite large.

**Figure 2:**
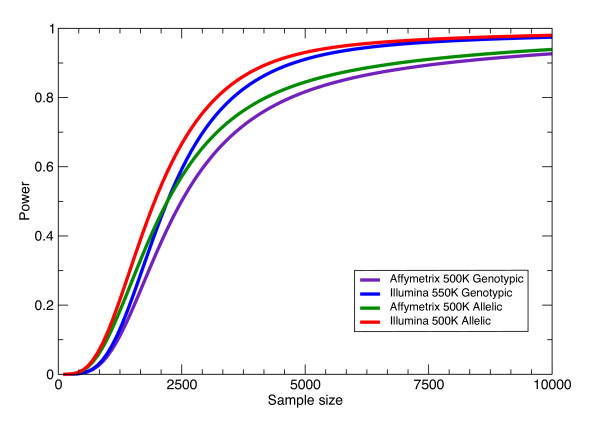
Power for genotypic and allelic tests. Data is shown for a GRR of 1.5 under a multiplicative model, the CEU HapMap population, and the specified genotyping system.

One critique of this approach is that the non-specific test used may not be the most powerful approach if we know the genetic model the disease follows. For instance, to study a trait that we believe follows a multiplicative model; a 2 × 2 contingency table to test for allelic association may be more appropriate. Power calculations for this test (Figure 2) shows that the relative pattern is the same as for a test of genotypic association, but the power is generally increased when an allelic test is used in instead of a genotypic test. Similar power calculations can be done if one wants to use an explicit test for a dominant or recessive mode of inheritance. However, as can be seen in this comparison between the Affymetrix 500 K and Illumina 550 K genotyping system, choice of SNPs and sample size can play a bigger role in determining power than choice of test. For the specified GRR of 1.5, the Illumina 550 K system with a genotypic test is more powerful than the Affymetrix 500 K system when sample size is greater than 2000 individuals (Figure 2).

**Figure 3:**
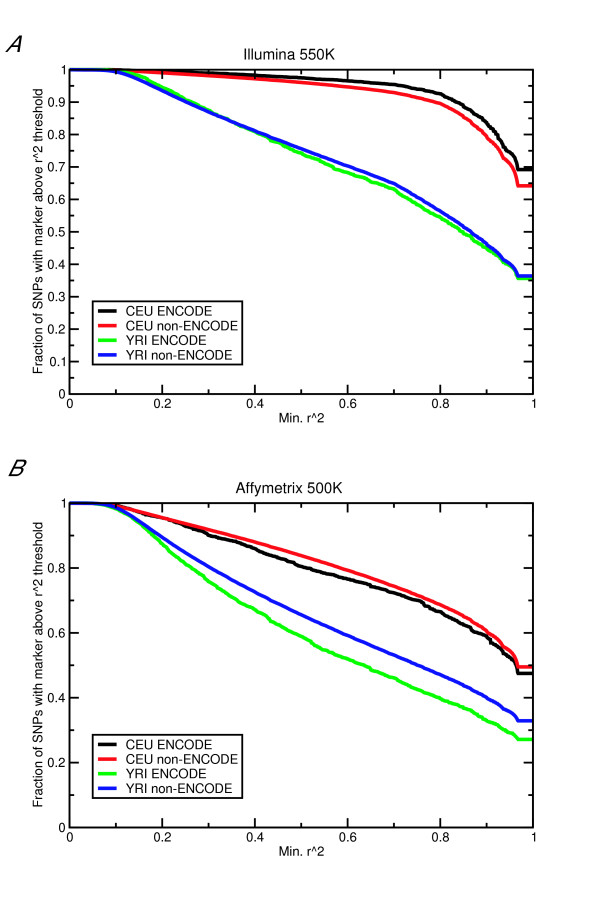
Coverage of tag SNPs. Fraction of non-tag SNPs in LD with a tag SNP with r^2 ^above specified threshold for the ENCODE and non-ENCODE regions of the HapMap project for the CEU and YRI populations. Results are shown for the Illumina 550 K **(A) **and Affymetrix 500 K **(B) **chips. The JPT+CHB population was not included because the curves generally overlap with the CEU curves and would make the graph harder to read. Results for the JPT+CHB population and for the other chips are qualitatively similar to the curves shown here.

Another possible criticism of this method is that the SNPs genotyped as part of the International HapMap Project may not be a representative subset of the common SNPs in the genome as a whole. To investigate this possibility, I compared the coverage of the various SNPs in the ENCODE and non-ENCODE regions from the HapMap project (Figure 3). Since the ENCODE regions of the HapMap project were completely resequenced in a subset of 48 individuals, I hypothesized that almost all common (minor allele frequency >5%) variants would have been identified in that region. If the SNPs genotyped as part of the HapMap are a representative subset of all of the common SNPs, then the coverage of an arbitrary set of tag SNPs should be equal for the two data sets. Assuming tag SNPs were chosen similarly for the ENCODE and non-ENCODE regions, relying on the HapMap data slightly overestimates r^2 ^with the tag SNPs and therefore could slightly inflate the power estimation. As the fraction of SNPs with an r^2 ^greater than the cutoff differs between the ENCODE and non-ENCODE regions by at most ten percentage points, and an average of three percentage points, this overestimation is not likely to be extreme.

**Figure 4:**
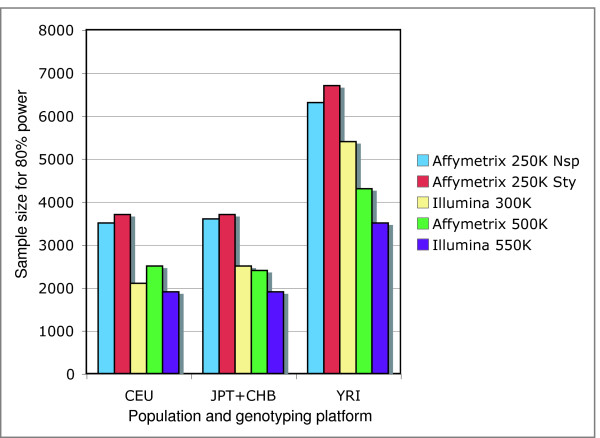
Total individuals required for 80% power. The computations assume the number of cases equals the number of controls and a GRR of 1.75. CEU, JPT+CHB, and YRI are the HapMap populations. Affy 250 K Nsp and Affy 250 K Sty represent the two chips that make up the Affymetrix 500 K genotyping system.

**Figure 5:**
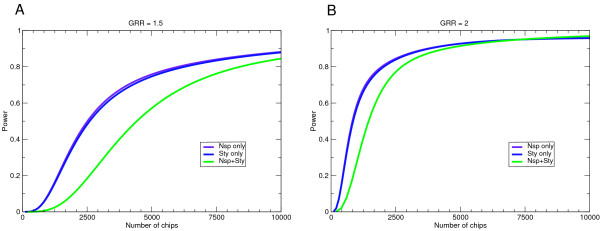
Power as a function of number of chips needed for the Affymetrix 500 K system and its two components. Calculations are done for a GRR of **(A) **1.5 and **(B) **2.0.

An easy and useful way to compare the power of different tag SNP sets in different populations is the sample size needed to achieve 80% power. The Illumina 550 K clearly performs best in all three populations (Figure 4). For the CEU population, the Illumina 300 K outperforms the Affymetrix 500 K, while in the other two populations the Affymetrix 500 K is better. This is not surprising, as the Illumina chips were optimized on CEU HapMap data. As the Affymetrix 500 K set is really two independent 250 K sets, I also looked at the power of each 250 K set individually. While the complete 500 K set of SNPs has more power than either half, the number of individuals required for 80% power using one half of the set is never twice the number required for the full set. This means that in cases when the number of chips that can be run rather than number of available samples is the limiting factor, it might make more sense to genotype more individuals using only one chip than to genotype fewer individuals using both chips. To test this hypothesis, I plotted power versus the number chips needed for the components of the Affymetrix 500 K system (Figure 5). The number of chips is simply the sample size for Nsp and Sty alone, and twice the sample size for the Nsp+Sty combination. Except in cases where power gets very high due to a large GRR and/or sample size, for a constant number of chips using only one of Nsp or Sty on more individuals provides a more powerful study.

I have presented a method to compute the power of a genome-wide association study in which a fixed set of tag SNPs will be genotyped. For the sake of simplicity, I only considered one straightforward single-SNP analysis scheme. While this approach has been used successfully [6], others have suggested that greater power can be obtained by looking at multiple tags or haplotypes [18,20]. This method for computing power can be adapted to such strategies provided it is possible to compute the power of detecting each SNP in the population given the set of tagging SNPs. I also assume that each SNP is equally likely to be functional. If we knew *a priori *the probability that a given SNP is functional, we could use this to weight the average power over all the SNPs. Such a weighting scheme would prioritize SNPs more likely to be of interest because of either functional considerations or location [21]. For instance, assume we assigned each SNP a probability of being the causative SNP based on external evidence such as a prior linkage study. If these probabilities are normalized to sum to one, they can be used to compute a weighted average power in this approach.

## Conclusion

Proper design of a genome-wide association study requires careful calculation of the power. These calculations will be invaluable to anyone who is planning a genome-wide association study. Using these calculations, the proper sample size to get adequate power in a given study can be computed. Furthermore, the performance of different genotyping platforms can be compared, allowing an investigator to choose whatever is best for his or her study. By performing such calculations, genome-wide association studies can be optimized to get the maximal power possible for a given set of resources.

## Methods

### Genotype data and populations

I used genotype data from release 21 (phase II) of the International HapMap project [19]. I used data from all four populations studied in the HapMap project. These populations are defined by the HapMap project as follows: Yoruba in Ibadan, Nigeria (abbreviation: YRI); Japanese in Tokyo, Japan (abbreviation: JPT); Han Chinese in Beijing, China (abbreviation: CHB); and CEPH (Utah residents with ancestry from northern and western Europe) (abbreviation: CEU). Similar to the analysis performed by the HapMap project, I combined genotypes from the JPT and CHB populations to make a joint JPT+CHB population. For all three resulting populations, I removed SNPs that have a minor allele frequency (MAF) less than 0.05 in that population. The remaining SNPs are considered to be "common." A summary of the number of SNPs remaining for each population is found in Table 1. When phased data is needed, I used the phased chromosomes for release 21.

### Calculation of power

To compute the overall power of an association study, I use three steps. First, I find the best tag SNP for each genotyped SNP in the data set. Then, I compute the power for each SNP assuming the specified GRR and sample size. Finally, I take an average power over all the SNPs to get the overall power.

To find the best tag SNP for each genotyped SNP, I look at the linkage disequilibrium between each SNP and all tag SNPs within 300 kb of it. For each pair of SNPs, I infer the two-locus haplotype frequencies between them using expectation maximization and compute r^2 ^between the two SNPs from the inferred haplotype frequencies [12]. The best tag is then taken to be the tag SNP with the highest value of r^2^.

To compute the power for a SNP, I assume that we are looking at genotype frequency differences using a two-degree of freedom *χ*^2 ^test. The power of this test is computed using a non-central *χ*^2 ^distribution with non-centrality parameter *λ*. Equations for *λ *have been previously derived for a general *χ*^2 ^test [22] and for application to genetic association [23]. Specifically, for genotypic association *λ *is given by:

λ=NANU[(p00−p10)2NAp00+NUp10+(p01−p11)2NAp01+NUp11+(p02−p12)2NAp02+NUp12]
 MathType@MTEF@5@5@+=feaafiart1ev1aaatCvAUfKttLearuWrP9MDH5MBPbIqV92AaeXatLxBI9gBaebbnrfifHhDYfgasaacH8akY=wiFfYdH8Gipec8Eeeu0xXdbba9frFj0=OqFfea0dXdd9vqai=hGuQ8kuc9pgc9s8qqaq=dirpe0xb9q8qiLsFr0=vr0=vr0dc8meaabaqaciaacaGaaeqabaqabeGadaaakeaaiiGacqWF7oaBcqGH9aqpcqWGobGtdaWgaaWcbaGaemyqaeeabeaakiabd6eaonaaBaaaleaacqWGvbqvaeqaaOGaei4waS1aaSaaaeaacqGGOaakcqWGWbaCdaWgaaWcbaGaeGimaaJaeGimaadabeaakiabgkHiTiabdchaWnaaBaaaleaacqaIXaqmcqaIWaamaeqaaOGaeiykaKYaaWbaaSqabeaacqaIYaGmaaaakeaacqWGobGtdaWgaaWcbaGaemyqaeeabeaakiabdchaWnaaBaaaleaacqaIWaamcqaIWaamaeqaaOGaey4kaSIaemOta40aaSbaaSqaaiabdwfavbqabaGccqWGWbaCdaWgaaWcbaGaeGymaeJaeGimaadabeaaaaGccqGHRaWkdaWcaaqaaiabcIcaOiabdchaWnaaBaaaleaacqaIWaamcqaIXaqmaeqaaOGaeyOeI0IaemiCaa3aaSbaaSqaaiabigdaXiabigdaXaqabaGccqGGPaqkdaahaaWcbeqaaiabikdaYaaaaOqaaiabd6eaonaaBaaaleaacqWGbbqqaeqaaOGaemiCaa3aaSbaaSqaaiabicdaWiabigdaXaqabaGccqGHRaWkcqWGobGtdaWgaaWcbaGaemyvaufabeaakiabdchaWnaaBaaaleaacqaIXaqmcqaIXaqmaeqaaaaakiabgUcaRmaalaaabaGaeiikaGIaemiCaa3aaSbaaSqaaiabicdaWiabikdaYaqabaGccqGHsislcqWGWbaCdaWgaaWcbaGaeGymaeJaeGOmaidabeaakiabcMcaPmaaCaaaleqabaGaeGOmaidaaaGcbaGaemOta40aaSbaaSqaaiabdgeabbqabaGccqWGWbaCdaWgaaWcbaGaeGimaaJaeGOmaidabeaakiabgUcaRiabd6eaonaaBaaaleaacqWGvbqvaeqaaOGaemiCaa3aaSbaaSqaaiabigdaXiabikdaYaqabaaaaOGaeiyxa0faaa@7F97@

where *N*_*A *_and *N*_*U *_are the number of case (affected) and control (unaffected) individuals, respectively; *p*_00_, *p*_01_, and *p*_02 _are the genotype frequencies in the cases; and *p*_10_, *p*_11_, and *p*_12 _are the genotype frequencies in the controls. If, instead of a 3 × 2 table we use a 2 × 2 table for a one-degree of freedom test of allelic association, the non-centrality parameter is given by

λ=2NANU(pA−pU)2NA+NU(NApA+NUpU)(NA+NU−NApA−NUpU)
 MathType@MTEF@5@5@+=feaafiart1ev1aaatCvAUfKttLearuWrP9MDH5MBPbIqV92AaeXatLxBI9gBaebbnrfifHhDYfgasaacH8akY=wiFfYdH8Gipec8Eeeu0xXdbba9frFj0=OqFfea0dXdd9vqai=hGuQ8kuc9pgc9s8qqaq=dirpe0xb9q8qiLsFr0=vr0=vr0dc8meaabaqaciaacaGaaeqabaqabeGadaaakeaaiiGacqWF7oaBcqGH9aqpcqaIYaGmcqWGobGtdaWgaaWcbaGaemyqaeeabeaakiabd6eaonaaBaaaleaacqWGvbqvaeqaaOGaeiikaGIaemiCaa3aaSbaaSqaaiabdgeabbqabaGccqGHsislcqWGWbaCdaWgaaWcbaGaemyvaufabeaakiabcMcaPmaaCaaaleqabaGaeGOmaidaaOWaaSaaaeaacqWGobGtdaWgaaWcbaGaemyqaeeabeaakiabgUcaRiabd6eaonaaBaaaleaacqWGvbqvaeqaaaGcbaGaeiikaGIaemOta40aaSbaaSqaaiabdgeabbqabaGccqWGWbaCdaWgaaWcbaGaemyqaeeabeaakiabgUcaRiabd6eaonaaBaaaleaacqWGvbqvaeqaaOGaemiCaa3aaSbaaSqaaiabdwfavbqabaGccqGGPaqkcqGGOaakcqWGobGtdaWgaaWcbaGaemyqaeeabeaakiabgUcaRiabd6eaonaaBaaaleaacqWGvbqvaeqaaOGaeyOeI0IaemOta40aaSbaaSqaaiabdgeabbqabaGccqWGWbaCdaWgaaWcbaGaemyqaeeabeaakiabgkHiTiabd6eaonaaBaaaleaacqWGvbqvaeqaaOGaemiCaa3aaSbaaSqaaiabdwfavbqabaGccqGGPaqkaaaaaa@6553@

where *p*_*A *_and *p*_*U *_are the frequencies of allele 0 in the cases and controls, respectively.

I use the Bonferroni correction for multiple testing and require a *p*-value of 0.05/M (where M is the number of tag SNPs genotyped) for statistical significance. When association is directly tested (the SNP is a tag SNP), I use the actual number of cases and controls to compute the power. For indirect association (the SNP is in LD with a tag SNP), I reduce the number of cases and controls by a factor of r^2 ^for the power computation [2].

I assume that the disease has a low enough prevalence in the population that the risk allele frequency in those without the disease approximates the risk allele frequency in the population. I can set the disease to follow a multiplicative, additive, dominant, or recessive model with a specified genotype relative risk (GRR) for the SNP of interest [1]. Given that genotype 0 is the wildtype, and taking *p*_10_, *p*_11_, and *p*_12 _from the observed genotype frequencies in the population, *p*_00_, *p*_01_, and *p*_02 _are computed as follows:

Multiplicative

p00=p102p11p12γ2p10p11+γp10p12+p102p11p12p01=γp10p12γ2p10p11+γp10p12+p102p11p12p02=γ2p10p11γ2p10p11+γp10p12+p102p11p12
 MathType@MTEF@5@5@+=feaafiart1ev1aaatCvAUfKttLearuWrP9MDH5MBPbIqV92AaeXatLxBI9gBaebbnrfifHhDYfgasaacH8akY=wiFfYdH8Gipec8Eeeu0xXdbba9frFj0=OqFfea0dXdd9vqai=hGuQ8kuc9pgc9s8qqaq=dirpe0xb9q8qiLsFr0=vr0=vr0dc8meaabaqaciaacaGaaeqabaqabeGadaaakeaafaqaaeWabaaabaGaemiCaa3aaSbaaSqaaiabicdaWiabicdaWaqabaGccqGH9aqpdaWcbaWcbaWaaSaaaeaacqWGWbaCdaqhaaadbaGaeGymaeJaeGimaadabaGaeGOmaidaaaWcbaGaemiCaa3aaSbaaWqaaiabigdaXiabigdaXaqabaWccqWGWbaCdaWgaaadbaGaeGymaeJaeGOmaidabeaaaaaaleaaiiGacqWFZoWzdaahaaadbeqaaiabikdaYaaalmaalaaabaGaemiCaa3aaSbaaWqaaiabigdaXiabicdaWaqabaaaleaacqWGWbaCdaWgaaadbaGaeGymaeJaeGymaedabeaaaaWccqGHRaWkcqWFZoWzdaWcaaqaaiabdchaWnaaBaaameaacqaIXaqmcqaIWaamaeqaaaWcbaGaemiCaa3aaSbaaWqaaiabigdaXiabikdaYaqabaaaaSGaey4kaSYaaSaaaeaacqWGWbaCdaqhaaadbaGaeGymaeJaeGimaadabaGaeGOmaidaaaWcbaGaemiCaa3aaSbaaWqaaiabigdaXiabigdaXaqabaWccqWGWbaCdaWgaaadbaGaeGymaeJaeGOmaidabeaaaaaaaaGcbaGaemiCaa3aaSbaaSqaaiabicdaWiabigdaXaqabaGccqGH9aqpdaWcbaWcbaGae83SdC2aaSaaaeaacqWGWbaCdaWgaaadbaGaeGymaeJaeGimaadabeaaaSqaaiabdchaWnaaBaaameaacqaIXaqmcqaIYaGmaeqaaaaaaSqaaiab=n7aNnaaCaaameqabaGaeGOmaidaaSWaaSaaaeaacqWGWbaCdaWgaaadbaGaeGymaeJaeGimaadabeaaaSqaaiabdchaWnaaBaaameaacqaIXaqmcqaIXaqmaeqaaaaaliabgUcaRiab=n7aNnaalaaabaGaemiCaa3aaSbaaWqaaiabigdaXiabicdaWaqabaaaleaacqWGWbaCdaWgaaadbaGaeGymaeJaeGOmaidabeaaaaWccqGHRaWkdaWcaaqaaiabdchaWnaaDaaameaacqaIXaqmcqaIWaamaeaacqaIYaGmaaaaleaacqWGWbaCdaWgaaadbaGaeGymaeJaeGymaedabeaaliabdchaWnaaBaaameaacqaIXaqmcqaIYaGmaeqaaaaaaaaakeaacqWGWbaCdaWgaaWcbaGaeGimaaJaeGOmaidabeaakiabg2da9maaleaaleaacqWFZoWzdaahaaadbeqaaiabikdaYaaalmaalaaabaGaemiCaa3aaSbaaWqaaiabigdaXiabicdaWaqabaaaleaacqWGWbaCdaWgaaadbaGaeGymaeJaeGymaedabeaaaaaaleaacqWFZoWzdaahaaadbeqaaiabikdaYaaalmaalaaabaGaemiCaa3aaSbaaWqaaiabigdaXiabicdaWaqabaaaleaacqWGWbaCdaWgaaadbaGaeGymaeJaeGymaedabeaaaaWccqGHRaWkcqWFZoWzdaWcaaqaaiabdchaWnaaBaaameaacqaIXaqmcqaIWaamaeqaaaWcbaGaemiCaa3aaSbaaWqaaiabigdaXiabikdaYaqabaaaaSGaey4kaSYaaSaaaeaacqWGWbaCdaqhaaadbaGaeGymaeJaeGimaadabaGaeGOmaidaaaWcbaGaemiCaa3aaSbaaWqaaiabigdaXiabigdaXaqabaWccqWGWbaCdaWgaaadbaGaeGymaeJaeGOmaidabeaaaaaaaaaaaaa@B83E@

Additive

p00=p102γp12+γp11+p10p01=γp112γp12+γp11+p10p02=2γp122γp12+γp11+p10
 MathType@MTEF@5@5@+=feaafiart1ev1aaatCvAUfKttLearuWrP9MDH5MBPbIqV92AaeXatLxBI9gBaebbnrfifHhDYfgasaacH8akY=wiFfYdH8Gipec8Eeeu0xXdbba9frFj0=OqFfea0dXdd9vqai=hGuQ8kuc9pgc9s8qqaq=dirpe0xb9q8qiLsFr0=vr0=vr0dc8meaabaqaciaacaGaaeqabaqabeGadaaakeaafaqabeWabaaabaGaemiCaa3aaSbaaSqaaiabicdaWiabicdaWaqabaGccqGH9aqpdaWcbaWcbaGaemiCaa3aaSbaaWqaaiabigdaXiabicdaWaqabaaaleaacqaIYaGmiiGacqWFZoWzcqWGWbaCdaWgaaadbaGaeGymaeJaeGOmaidabeaaliabgUcaRiab=n7aNjabdchaWnaaBaaameaacqaIXaqmcqaIXaqmaeqaaSGaey4kaSIaemiCaa3aaSbaaWqaaiabigdaXiabicdaWaqabaaaaaGcbaGaemiCaa3aaSbaaSqaaiabicdaWiabigdaXaqabaGccqGH9aqpdaWcbaWcbaGae83SdCMaemiCaa3aaSbaaWqaaiabigdaXiabigdaXaqabaaaleaacqaIYaGmcqWFZoWzcqWGWbaCdaWgaaadbaGaeGymaeJaeGOmaidabeaaliabgUcaRiab=n7aNjabdchaWnaaBaaameaacqaIXaqmcqaIXaqmaeqaaSGaey4kaSIaemiCaa3aaSbaaWqaaiabigdaXiabicdaWaqabaaaaaGcbaGaemiCaa3aaSbaaSqaaiabicdaWiabikdaYaqabaGccqGH9aqpdaWcbaWcbaGaeGOmaiJae83SdCMaemiCaa3aaSbaaWqaaiabigdaXiabikdaYaqabaaaleaacqaIYaGmcqWFZoWzcqWGWbaCdaWgaaadbaGaeGymaeJaeGOmaidabeaaliabgUcaRiab=n7aNjabdchaWnaaBaaameaacqaIXaqmcqaIXaqmaeqaaSGaey4kaSIaemiCaa3aaSbaaWqaaiabigdaXiabicdaWaqabaaaaaaaaaa@7AC7@

Dominant

p00=p10γp12+γp11+p10p01=γp11γp12+γp11+p10p02=γp12γp12+γp11+p10
 MathType@MTEF@5@5@+=feaafiart1ev1aaatCvAUfKttLearuWrP9MDH5MBPbIqV92AaeXatLxBI9gBaebbnrfifHhDYfgasaacH8akY=wiFfYdH8Gipec8Eeeu0xXdbba9frFj0=OqFfea0dXdd9vqai=hGuQ8kuc9pgc9s8qqaq=dirpe0xb9q8qiLsFr0=vr0=vr0dc8meaabaqaciaacaGaaeqabaqabeGadaaakeaafaqaaeWabaaabaGaemiCaa3aaSbaaSqaaiabicdaWiabicdaWaqabaGccqGH9aqpdaWcbaWcbaGaemiCaa3aaSbaaWqaaiabigdaXiabicdaWaqabaaaleaaiiGacqWFZoWzcqWGWbaCdaWgaaadbaGaeGymaeJaeGOmaidabeaaliabgUcaRiab=n7aNjabdchaWnaaBaaameaacqaIXaqmcqaIXaqmaeqaaSGaey4kaSIaemiCaa3aaSbaaWqaaiabigdaXiabicdaWaqabaaaaaGcbaGaemiCaa3aaSbaaSqaaiabicdaWiabigdaXaqabaGccqGH9aqpdaWcbaWcbaGae83SdCMaemiCaa3aaSbaaWqaaiabigdaXiabigdaXaqabaaaleaacqWFZoWzcqWGWbaCdaWgaaadbaGaeGymaeJaeGOmaidabeaaliabgUcaRiab=n7aNjabdchaWnaaBaaameaacqaIXaqmcqaIXaqmaeqaaSGaey4kaSIaemiCaa3aaSbaaWqaaiabigdaXiabicdaWaqabaaaaaGcbaGaemiCaa3aaSbaaSqaaiabicdaWiabikdaYaqabaGccqGH9aqpdaWcbaWcbaGae83SdCMaemiCaa3aaSbaaWqaaiabigdaXiabikdaYaqabaaaleaacqWFZoWzcqWGWbaCdaWgaaadbaGaeGymaeJaeGOmaidabeaaliabgUcaRiab=n7aNjabdchaWnaaBaaameaacqaIXaqmcqaIXaqmaeqaaSGaey4kaSIaemiCaa3aaSbaaWqaaiabigdaXiabicdaWaqabaaaaaaaaaa@76FE@

Recessive

p00=p10γp12+p11+p10p01=p11γp12+p11+p10p02=γp12γp12+p11+p10
 MathType@MTEF@5@5@+=feaafiart1ev1aaatCvAUfKttLearuWrP9MDH5MBPbIqV92AaeXatLxBI9gBaebbnrfifHhDYfgasaacH8akY=wiFfYdH8Gipec8Eeeu0xXdbba9frFj0=OqFfea0dXdd9vqai=hGuQ8kuc9pgc9s8qqaq=dirpe0xb9q8qiLsFr0=vr0=vr0dc8meaabaqaciaacaGaaeqabaqabeGadaaakeaafaqabeWabaaabaGaemiCaa3aaSbaaSqaaiabicdaWiabicdaWaqabaGccqGH9aqpdaWcbaWcbaGaemiCaa3aaSbaaWqaaiabigdaXiabicdaWaqabaaaleaaiiGacqWFZoWzcqWGWbaCdaWgaaadbaGaeGymaeJaeGOmaidabeaaliabgUcaRiabdchaWnaaBaaameaacqaIXaqmcqaIXaqmaeqaaSGaey4kaSIaemiCaa3aaSbaaWqaaiabigdaXiabicdaWaqabaaaaaGcbaGaemiCaa3aaSbaaSqaaiabicdaWiabigdaXaqabaGccqGH9aqpdaWcbaWcbaGaemiCaa3aaSbaaWqaaiabigdaXiabigdaXaqabaaaleaacqWFZoWzcqWGWbaCdaWgaaadbaGaeGymaeJaeGOmaidabeaaliabgUcaRiabdchaWnaaBaaameaacqaIXaqmcqaIXaqmaeqaaSGaey4kaSIaemiCaa3aaSbaaWqaaiabigdaXiabicdaWaqabaaaaaGcbaGaemiCaa3aaSbaaSqaaiabicdaWiabikdaYaqabaGccqGH9aqpdaWcbaWcbaGae83SdCMaemiCaa3aaSbaaWqaaiabigdaXiabikdaYaqabaaaleaacqWFZoWzcqWGWbaCdaWgaaadbaGaeGymaeJaeGOmaidabeaaliabgUcaRiabdchaWnaaBaaameaacqaIXaqmcqaIXaqmaeqaaSGaey4kaSIaemiCaa3aaSbaaWqaaiabigdaXiabicdaWaqabaaaaaaaaaa@7077@

After the power is computed for each SNP, I take the overall power to be the average power over all the SNPs. In taking the average power over all SNPs, I give less weight to the tag SNPs since they are over-represented in the set of SNPs being analyzed. Assume that of the *S *SNPs under consideration (for which we have linkage disequilibrium [LD] data from, for instance, the HapMap project), *M *are tags that will be genotyped on the chip and *S-M *are not tags. Further assume that there are *T *common SNPs in total in this population, which includes both those *S *SNPs for which we have LD data and SNPs for which we do not know their LD with surrounding SNPs. Let 1-*β*_*i *_be the power for SNP *i *where *i *ranges from 1 to *S *and SNP *i *is a tag SNP when *i *≤ *M *and a non-tag otherwise. Then, the overall power is given by:

Power=∑i=1M[1T(1−βi)]+∑i=M+1S[T−MT(S−M)(1−βi)]
 MathType@MTEF@5@5@+=feaafiart1ev1aaatCvAUfKttLearuWrP9MDH5MBPbIqV92AaeXatLxBI9gBaebbnrfifHhDYfgasaacH8akY=wiFfYdH8Gipec8Eeeu0xXdbba9frFj0=OqFfea0dXdd9vqai=hGuQ8kuc9pgc9s8qqaq=dirpe0xb9q8qiLsFr0=vr0=vr0dc8meaabaqaciaacaGaaeqabaqabeGadaaakeaacqWGqbaucqWGVbWBcqWG3bWDcqWGLbqzcqWGYbGCcqGH9aqpdaaeWbqaamaadmaabaWaaSaaaeaacqaIXaqmaeaacqWGubavaaGaeiikaGIaeGymaeJaeyOeI0ccciGae8NSdi2aaSbaaSqaaiabdMgaPbqabaGccqGGPaqkaiaawUfacaGLDbaaaSqaaiabdMgaPjabg2da9iabigdaXaqaaiabd2eanbqdcqGHris5aOGaey4kaSYaaabCaeaadaWadaqaamaalaaabaGaemivaqLaeyOeI0Iaemyta0eabaGaemivaqLaeiikaGIaem4uamLaeyOeI0Iaemyta0KaeiykaKcaaiabcIcaOiabigdaXiabgkHiTiab=j7aInaaBaaaleaacqWGPbqAaeqaaOGaeiykaKcacaGLBbGaayzxaaaaleaacqWGPbqAcqGH9aqpcqWGnbqtcqGHRaWkcqaIXaqmaeaacqWGtbWua0GaeyyeIuoaaaa@61EE@

In this manner, the tag SNPs are only considered representative of themselves, while the non-tag SNPs for which we have LD data are considered representative of all common non-tag SNPs. For these calculations, I use *T *= 2 × 10^7^.

### Implementation

A computer program to implement these calculations was written in C. The source code is available upon request from the author.

## Authors' contributions

RJK conceived of the experiments, implemented them, analyzed the data, and wrote the manuscript.

## Supplementary Material

Additional file 1 Power of genome-wide association studies with various parameters. Each line of the file contains the power of a genome-wide association study conducted with the specified HapMap population, genetic model, and sample size (N) based on the SNPs present in a variety of commercially available genotyping products. Click here for file
